# Effects of 810 nm treatments in acute myofiber contraction of C2C12 myotubes

**DOI:** 10.1371/journal.pone.0327008

**Published:** 2025-06-27

**Authors:** Nashwa Cheema, Linh Pham, Alexa Nazarian, Namrata Ghag, Emma Wise, Christiane Fuchs, Richard Rox Anderson, Joshua Tam

**Affiliations:** 1 Wellman Center for Photomedicine, Massachusetts General Hospital, Boston, Massachusetts, United States of America; 2 Department of Dermatology, Harvard Medical School, Boston, Massachusetts, United States of America; 3 Harvard-Massachusetts Institute of Technology (MIT), Health Sciences and Technology (HST), Cambridge, Massachusetts, United States of America; University of Minnesota Medical School, UNITED STATES OF AMERICA

## Abstract

The muscoskeletal system can be irradiated with wavelengths in the red and near infrared regions which penetrate deep into the body and stimulate biological mechanisms. However, the activation of cellular responses in muscle, specifically actively contracting, is not clearly understood. Therefore, we investigated biological effects induced by irradiation with 810 nm wavelength of light in myotubes, resting or actively contracting in an acute model of exercise. In resting myotubes, cytosolic Ca^2+^ rose within 10 minutes post treatment with 810 nm at 2–4 J/cm^2^. ATP production was increased 4% ± 3 at 24 hrs post light treatment. In contracting myotubes, 810 nm treatment resulted in a significant ~30% increase in intracellular ATP levels and a 20% ± 12 reduction in lactate secretion into cell culture media. 810 nm treated myotubes also had a smaller change in myotube width during contractions, 5% ± 3, suggesting the myotubes were contracting with less force. Although the contractile motion was reduced, 810 nm treated myotubes had a higher frequency of spontaneous contractions after removal of electric pulse stimulation (EPS), 42% ± 21 and 1.3-2 – fold increase in mitochondrial proteins, Tom70, citrate synthase (CS) and succinate dehydrogenase (SDHA). This finding suggests that 810 nm treatment altered metabolic and contractile properties of myotubes due to mitochondrial activation. A more thorough understanding of these effects could lead to new treatment modalities that could improve physical performance.

## Introduction

Photobiomodulation (PBM) is the application of non-ionizing electromagnetic energy on tissues to stimulate endogenous chromophores to induce photochemical reactions that modulate biological responses. The effective fluence (energy per area) utilized is very low and there is minimal heat generation, making PBM a practical non-invasive therapeutic strategy. The mechanism of PBM is mediated through chromophores present in the cells that absorb energy from light and become activated. The primary mitochondrial chromophore believed to mediate PBM effects, is cytochrome c oxidase (COX), complex IV in the electron transport chain, that absorbs wavelengths from 600–900 nm and increases ATP production. Another putative mechanism is energy being absorbed, from longer wavelengths (> 900 nm), by water and light-sensitive ion channels which open upon activation and stimulate cellular responses affecting energy and oxidative stress homeostasis [1].

PBM has become a common practice in sports medicine and is used to reduce inflammation, muscular injuries, fatigue and prevent loss in performance [2,3]. Treatment has shown to improve muscle performance both in humans and rodent models. In situations of muscle disuse in rodent experimental models, PBM has reduced inflammation and attenuated muscle atrophy [4–7]. Skeletal muscle is a highly metabolic tissue that is enriched in mitochondria, therefore, responds to photobiomodulation. Most studies have used wavelengths in the red/near infrared spectrum (660,780,810 and 980 nm) and observed effects in increased muscle endurance, oxidative capacity and reduced inflammation [2,3]. However, the mechanism(s) of cellular responses by PBM in muscle is not clearly understood. There is no standardization in parameters for PBM therapy, with researchers and clinicians using a wide range of wavelengths, fluences, treatment duration/frequency, etc. in PBM applications.

NIR light treatments of C2C12 myotubes have increased key regulators of mitochondrial biogenesis [8] and mitochondrial ATP production [9]. However, the biological response of myotubes when resting is vastly different than when they are actively contracting [10,11]. C2C12 myotubes can be induced to contract via electrical pulse stimulation (EPS) to simulate skeletal muscle contractions occurring in exercise physiology [10,11] and are used as an *in vitro* model for exercise [10,12,13]. Electrically stimulated myotubes have a similar transcriptional response as seen in trained mice [14]. Myotubes visibly contract with the release of calcium into the cytoplasm [12]. Metabolic changes, e.g., change in ATP levels, glucose uptake and lactate release occur with electrical stimulation [13]. Hence in this study, we elucidate the cellular responses to 810 nm wavelength of light, in an acute model of exercise in myotubes.

## Materials and methods

### Cell culture

C2C12 (ATCC CRL-1772) myoblasts are murine muscle progenitor cells that differentiate into myotubes when growth media is supplemented with 5% horse serum [15]. Myoblasts were grown in high glucose DMEM supplemented with 10% FBS and 1% penicillin and streptomycin. 20,000 cells/ml were seeded in 24 cell culture plates and allowed to proliferate to 90% confluency. Growth media was switched to differentiation media which consisted of 5% inactivated horse serum and 1% PenStrep in DMEM. Differentiation media was changed every other day and all experiments were performed when myotubes reached Day 5/6 of differentiation.

### Irradiation of cells

For irradiation treatments, we built a customized LED device for 24-well cell culture plates, modified from a previously developed open-source LED-based platform by Gerhardt et. al. [16]. Cell culture plates were placed on top of the device and treated with 810 LED at 37ºC at various parameters outlined in Table 1. The LED sources were pulsed at 3.8kHz and the average power output was detected by a photodiode sensor (Ophir, PD300) presented in Table 1.

**Table 1:** 810 nm Light parameters.

Fluence J/cm^2^	Time min	Power mW	Irradiance mW/cm^2^
0.5	1	6.5	8.3
1	2	6.5	8.3
2	4	6.5	8.3
4	8	6.5	8.3

### Ca^2+^ and ATP

Cytosolic levels of Ca^2+^ was measured by fluorescent dye Fura-2 (Invitrogen Cat # F1225). Cells were prestained with 1 µM Fura-2 prepared in DMEM for 45 mins at 37°C 5% CO_2_ incubator prior to NIR treatment. After treatment, cells were allowed to sit at room temperature for 10 mins and the fluorescent intensity was measured in the plate reader (SpectraMax M5) with 2 excitation wavelengths 340 nm and 380 nm with emission at 510 nm. The ratio of fluorescent intensity at 340 nm and 380 nm was calculated to determine Ca^2+^ intracellular levels. Intracellular ATP was measured using CellTiter-Glo kit (Promega Cat # G9242) following the manufacturer’s instructions.

### DCF fluorescence

C2C12 myotubes were incubated with 10 µM H_2_DCFDA (ThermoFisher Cat# D399) in DMEM for 20 mins in the 37°C incubator. After the incubation period, cells were washed with PBS and 1 ml of HBSS was added to the wells. The nonfluorescent H_2_DCFDA is converted to a fluorescent end product 2’,7’-dichlorofluorescein (DCF) in the presence of oxidative stress. To measure fluorescent intensity, wells were imaged in SpectraMax M5 microplate reader at 495 nm excitation and 525 nm emission. To induce oxidative stress, cells were incubated with 100 µM hydrogen peroxide, H_2_O_2_ (SigmaAldrich Cat# 7722841) for 10mins at room temperature. To inhibit oxidative stress, cells were preincubated with 500 µM N-acetylcysteine (NAC) (SigmaAldrich Cat# A7250) in media for 6hrs in the 37°C 5% CO_2_ incubator.

### Electrical pulse stimulation (EPS)

Carbon electrodes (C-Dishes) adapted to fit in a 24 well tissue culture dish were purchased from IonOptix Inc. Electrodes were placed under UV for 30 minutes prior to being immersed into cell culture well for sterilization. The stimulator was set at an electric pulse of 5V, frequency of 2 Hz with a pulse duration of 5 msec. After connecting the 24 well cell culture plate with the C-Dish to the stimulator, the cells were placed back in the 37°C incubator and allowed to contract for 3hrs. Immediately before EPS and 24hrs prior, three wells were irradiated with 810 nm at 4 J/cm^2^. 3–4 wells were used as non-PBM contracting controls.

### Biochemical assays after EPS

At the end of the 3hr EPS, electrodes were removed, and cell culture media was mixed with a 1 ml pipette to create a homogenous mixture of secreted lactate. The cells were used to measure glucose uptake, ATP, protein levels and cell culture media was used to measure lactate and LDH release. Lactate levels were immediately determined via the Lactate-Glo kit (Promega Cat # J5022) and LDH levels were measured via Cytotoxicity Detection Kit (LDH) (Roche Cat # 11644793001). Glucose uptake by the cells was measured via 2-deoxyglucose, 2-DG, a modified glucose molecule unable to be metabolized through glycolysis. Myotubes were incubated with 2-DG at room temperature after which cells were lysed and 2-DG levels were measured via the GlucoseUptake-Glo (Promega Cat# J1343). Cell lysates were also used to measure ATP and protein concentration with the Ionic Detergent Compatibility Reagent (ThermoScientific Cat # 22663).

### Live cell microscopy

Phase-contrast microscopy images were taken with time lapse imaging (Spot 5.3 software) selected at the shortest interval for ~50 frames. Imaging was performed to observe changes in contractile motion during electric stimulation and determine frequency of spontaneous contractions after removal of stimulation. The frequency was calculated by counting the total number of myotubes and myotubes that underwent at least 1 contraction in the imaging field in ImageJ [17]. Experiments were performed three times with 3 replicates/well per experiment. Changes in contractile motion were determined from a total of ~15 individual fibers per experimental group. Myotube width was measured in image J with the straight line tool. The ROI line segment was copied, when the fiber was relaxed, to the following consecutive frame, when the fiber contracted. The line segment was then shortened to the width of the myofiber. The ratio of contraction and pre-contraction measurement was calculated and subtracted from 1 to determine change in width. To observe calcium signaling during contractions, the cells were stained with Fluo-8 (Abcam Cat # ab142773) for 20 mins after 3hrs of contraction. The electrodes were placed back into the cell culture well to induce contractions which were imaged on a fluorescent microscope. Time lapse imaging (Spot 5.3 software) was selected at the shortest interval for ~20 frames. Imaging was taken for 3–5 contractions per well with a total of 3 wells per experimental group.

### Protein lysates and capillary immunoblots

Myotubes were washed with PBS and protein lysates were prepared by collecting cells in MPER Cell lysis buffer (ThermoFisher Cat # 78501) with protease and phosphatase inhibitor (ThermoScientific Cat #1862209). Protein concentration was measured via the absorbance of Bradford reagent (ThermoFisher). 1 µg of protein was loaded into the assay plate on 12–230 kDA Separation Modules (ProteinSimple SM W003). The primary antibody was used at the dilution of 1:20 for anti-rabbit Tom70 (Novus Biologicals Cat# NBP1-87863) and 1:1600 for anti-mouse SDH A (Abcam Cat# ab14715), with 1:20 anti-rabbit β-actin (Cell Signaling Cat #4970S) as loading control. For chemiluminescence detection the anti-rabbit detection module (ProteinSimple Cat # DM-001), anti-mouse detection module (ProteinSimple Cat # DM-002) and 20X anti-rabbit HRP conjugate (ProteinSimple Cat# 043-426) was used accordingly. Raw data is presented in S1 Fig and S1 File.

### Immunocytochemistry

Myotubes were induced to contract for 3hrs after which the electrodes were removed, and the cells were washed with PBS. 4% PFA (ThermoScientific Cat# 28906) was added to each well for 15mins followed by incubation with 0.03% Triton (Sigma Cat# X-100) for 15mins and 1% BSA in PBS (ThermoFisher Cat# 37525) for 30mins blocking at room temperature. The dilutions of the primary antibodies used was 1:500 for anti-rabbit citrate synthase (Abcam Cat# ab96600) and anti-mouse SDHA (Abcam Cat# ab14715). The secondary antibodies were anti-mouse IgG Alexa Fluor 555 (Abcam Cat # ab150114) and anti-rabbit IgG Alexa Fluor 488 (Abcam Cat# ab150077) at 1:500 dilution. The cells were imaged in Operetta (PerkinElmer) in 24 well cell culture plates, and images were taken at 10x objective with 1 well/ experimental group (EPS and 810 EPS). Experiment was replicated 4 times.

### Statistical analysis

**Fig 1:**
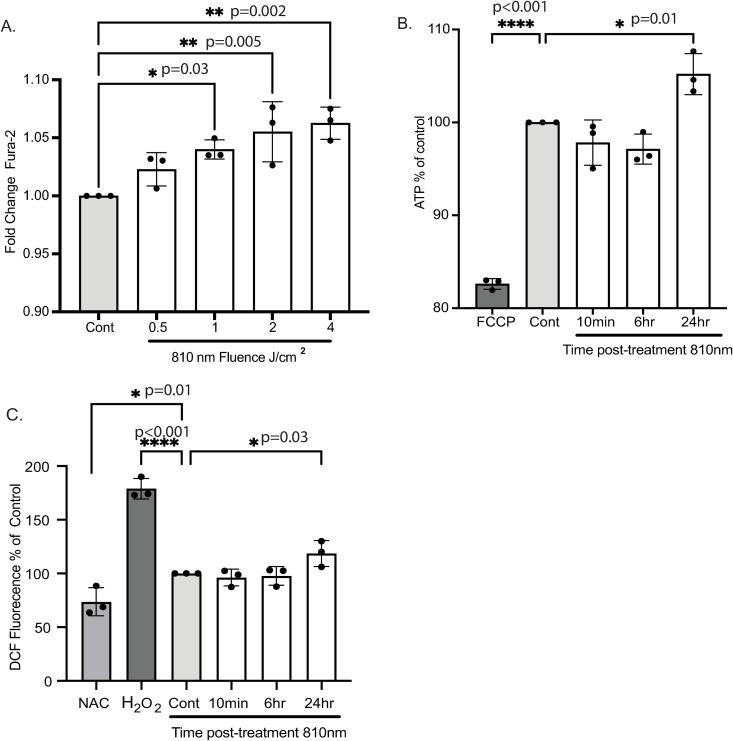
Intracellular levels of Ca2+, ATP and ROS increase with 810 nm treatment. (A) Four different fluences were tested with 810 nm. Cells were preincubated with Fura-2 calcium indicator dye, treated with the respective fluences, and allowed to sit at room temperature for 10 minutes before detection of fluorescence in the plate reader. Fold change was calculated per fluence. 2-4 J/cm^2^ of 810nm treatment were most significant. (B) ATP was measured for three time points, 10 mins, 6 hrs and 24 hrs post treatment with 810 nm at 4 J/cm^2^. At each time point, controls were present to normalize data. FCCP was used as an inhibitor of ATP synthesis as a negative control. At 24 hrs with 810 nm treatment, ATP levels were increased. (C) DCF fluorescence indicative of ROS generation in 810 nm treated myotubes at indicated time points. Three independent experiments were conducted with n = 3-6 wells per experiment. Average values ± SD for each experiment are represented with significance reported as *p-value < 0.05, **p-value < 0.01, *** p-value < 0.001, **** p-value < 0.0001.

**Fig 2:**
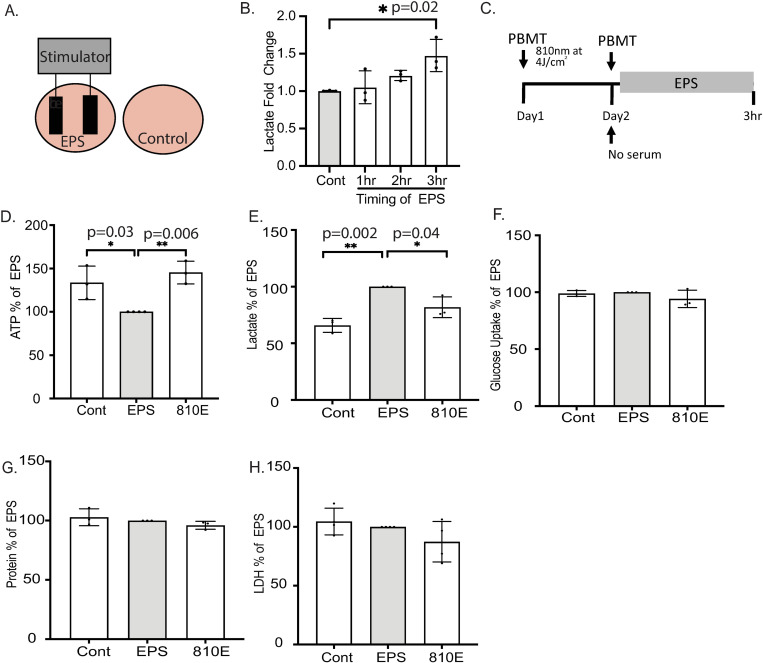
Pretreatments of electric pulse stimulated (EPS) myotubes with 810nm reduces lactate release. (A) Cells were stimulated to contract by immersing carbon electrodes in the cell culture media. A diagram depicting a control well and EPS well are shown. (B) Lactate levels in supernatant measured at 1, 2 and 3hr EPS treatment. (C) A schematic of experimental timeline is shown. Cells were stimulated to contract for 3hr. Prior to contraction, myotubes were treated with 810 nm 24hr earlier and immediately before the start of electric stimulation. All biochemical assays were performed immediately after 3hr of EPS (D) ATP levels in control, EPS and EPS with 810 nm treated cells. EPS myotubes had the least ATP levels and pretreatments with either NIR wavelength resulted in an increase. (E) Cell culture media was used to measure lactate levels. (F-H) Glucose uptake by cells, intracellular protein levels and LDH leak into cell culture media was measured. Three-four independent experiments were conducted with n = 3-6 wells per experiment. Average values ± SD for each experiment are represented with significance reported as *p-value < 0.05, **p-value < 0.01, *** p-value < 0.001, **** p-value < 0.0001.

**Fig 3:**
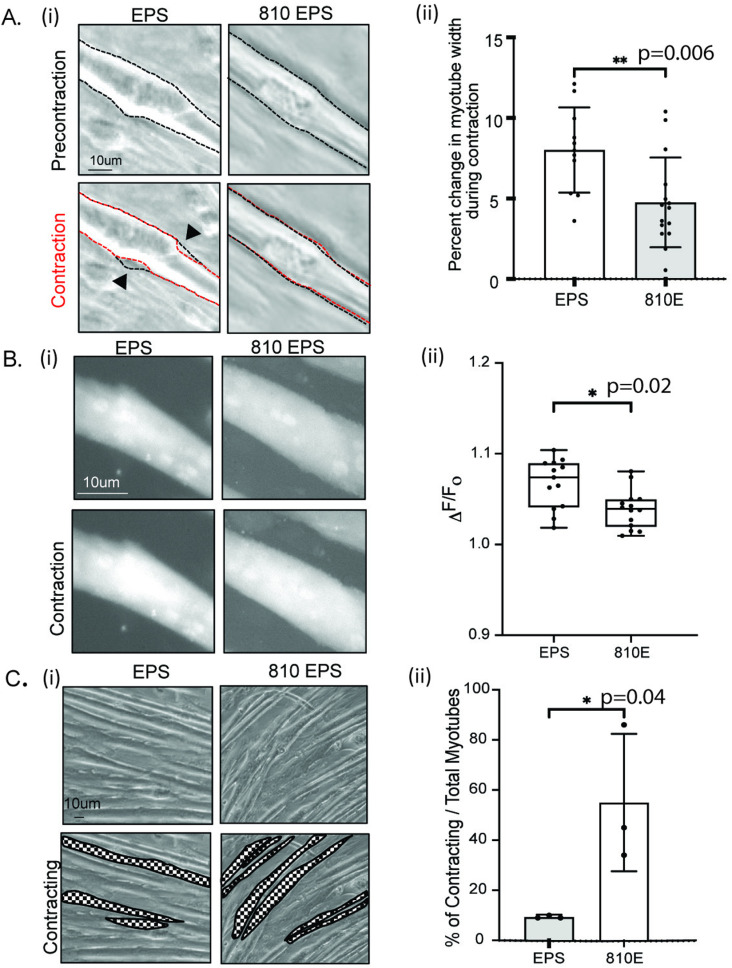
Electrically stimulated myotubes treated with 810 nm have reduced contractile motion, lower calcium and increase in spontaneous contractions. (A) i) Top panel depicts myotubes pre-contraction that are in a relaxed state. A resting myotube is traced in a dashed black line. The bottom panel depicts the same myotube during contraction. The contracting myotube is traced in red. The outline of the precontraction is overlayed on the contracting myotube to demonstrate myotube movement occurring upon contraction. EPS myotubes show larger movement upon contraction (arrow) whereas 810 nm pretreatment shows reduced/no change in contractile motion. ii) The width of individual myotubes was measured during contraction and precontraction in image J. Percent change in myotube width during contraction was measured for all experimental groups. Bar graphs represent mean ± SD. Three independent experiments were performed to achieve a total of 15-19 myotubes. (B) i) Myotubes were stained with Fluo-8, a calcium fluorescent probe and visualized under the microscope. Representative fluorescence images are shown ii) To measure change in fluorescence in individual myotubes, cytosolic portions of the myotubes that were not overexposed were selected to measure fluorescent intensity precontraction and contraction state of the myotube. The difference of intensity was measured for a total of 16 myotubes. Box plots represent data range with whiskers denoting minimum to maximum. (C) EPS myotubes were allowed to recover for 30 minutes. Myotubes were visualized under the microscope and image sequences were taken for each experimental group. The contracting myotubes have been outlined and shaded in the bottom panel for each group. ii) To measure frequency, total number of myotubes that were present and myotubes that were contracting in 4x objective view were counted. Three independent experiments were conducted with n = 3-6 wells per experiment. Average values ± SD for each experiment are represented with significance reported as *p-value < 0.05, **p-value < 0.01, *** p-value < 0.001, **** p-value < 0.0001.

**Fig 4:**
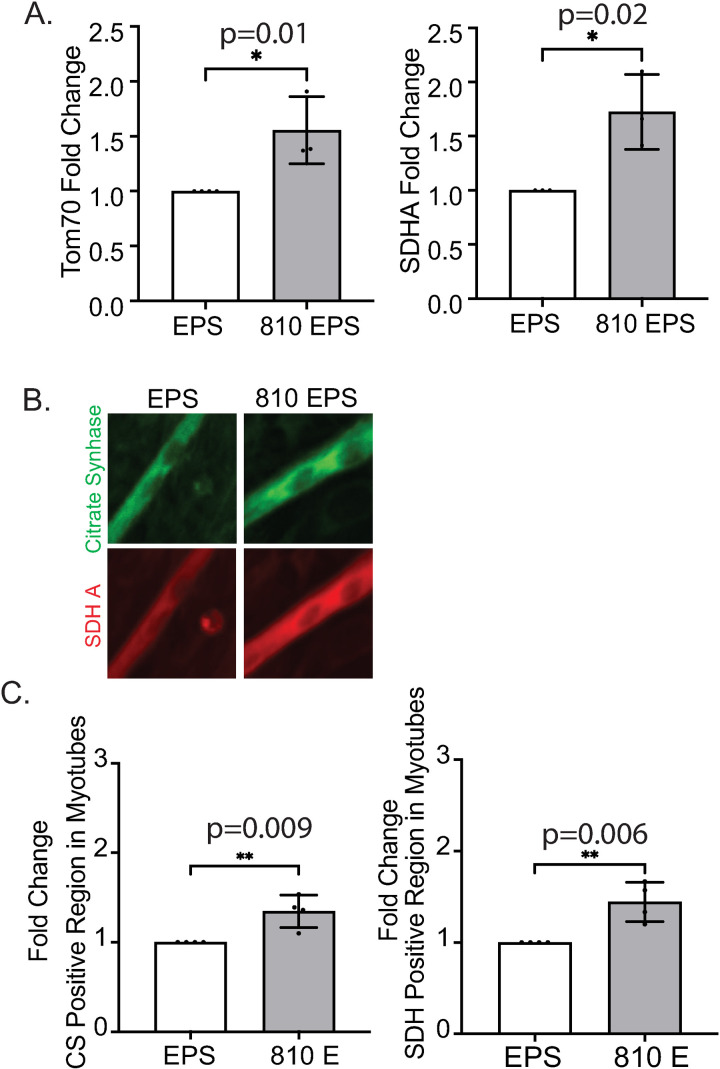
NIR light pretreatment in electrically pulsed myotubes alters mitochondrial protein expression. (A) Tom70 and SDH A protein expression was normalized to β-actin and fold change of NIR pretreatments to EPS was determined for the proteins. 4 independent experiments were performed for Tom70 and 3 independent experiments for SDHA. Raw data from the capillary western immune assay is presented in S1 Fig. (B) Myotubes were stained for Citrate Synthase (green), a mitochondrial matrix protein and SDHA (red). (C) The number of positive fibers for CS and SDH were normalized to number of myotubes in the objective field. Fold change, compared to EPS, was calculated for 810 E. Bar graphs represented as mean ± SD. 4 independent experiments were performed. Significance reported as *p-value < 0.05, **p-value < 0.01, *** p-value < 0.001, ****p-value < 0.0001.

All data is reported as mean ± SD. Multiple cell culture wells (3–5 wells) per experiment were averaged for a total of 3–4 independent experiments. One-way ANOVA with Bonferroni’s multiple comparison was performed for Figs 1 and 2. Student t-test were performed for Figs 3 and 4 when comparing 810 to EPS. For measurements of calcium release during myotube contraction (Fig 3B), the data was averaged for 3–5 contractions per myotube and a total of 16 myotubes were analyzed. Differences are considered statistically significant for p < 0.05. Statistical analyses were performed in GraphPad Prism version 8.0.0 for Windows, GraphPad Software, San Diego, California USA, www.graphpad.com.

## Results

### Intracellular levels of Ca^2+^, ATP and ROS increase with 810 nm treatment

Cytosolic calcium levels increase immediately upon changes to extracellular environments via cation channels on the cell membrane. One of these channels, transient receptor potential (TRP), is sensitive to light in the wavelength range of 800–1064 nm [18]. To assess whether 810 nm affected calcium signaling in myotubes, four different fluences were tested and the LED source parameters are listed in Table 1. After PBM treatment, cells were allowed to recover for 10 mins and intracellular calcium levels was quantified using a radiometric calcium indicator dye and normalized to non-PBM controls to determine the fold change difference. A significant increase of 6% ± 4 at 4 J/cm^2^ for 810 nm (Fig 1A), the highest fluence tested, when compared to controls was detected with irradiation treatment. For all the following experiments, we chose to use 4 J/cm^2^ for 810 nm treatments.

To determine mitochondrial response to light irradiation, we measured intracellular levels of ATP in a time course experiment at 10mins, 6 and 24 hours post PBM treatment. To inhibit ATP production, we used carbonyl cyanide p-trifluoro methoxyphenylhydrazone (FCCP) as a negative control to disrupt the membrane potential. We normalized all values to baseline control (non-PBM treated) to measure the percent change of ATP levels. 810 nm treatment resulted in a significant increase (4% ± 3) in ATP levels at 24h compared to controls (Fig 1B). Next, we assessed reactive oxygen species (ROS). ROS levels are associated with cellular respiration and ATP production. To support our ATP observation, we did a similar time course experiment to determine changes in intracellular ROS levels. We observed at 24hrs after PBM treatment 810 nm had 22% ± 28 increase in intracellular ROS when compared to controls, which parallels the changes in ATP levels (Fig 1C). We used H_2_O_2_ as a positive control to induce production of ROS and N-acetylcysteine (NAC) as a negative control which acts a scavenger for free intracellular ROS.

### In electric pulse stimulated (EPS) myotubes, ATP levels are maintained, and lactate secretion is reduced with 810nm pretreatment

C2C12 myotubes can be induced to contract by electric pulse stimulation (EPS) to mimic exercise. To test our hypothesis that NIR light can aid in energy metabolism during contraction, we stimulated myotubes with EPS. Carbon electrodes are immersed into the wells containing cell culture media and is connected to an external stimulator outside of the incubator as depicted in Fig 2A. During an intense bout of exercise, muscle enters anaerobic respiration where oxygen is limited for ATP generation and lactic acid is produced. We tested 1, 2 and 3hr duration of EPS for lactate secretion into the cell culture supernatant and observed a significant increase with 2 and 3hr duration of EPS with the highest fold change detected at 3hrs of EPS when compared to non-contracting (resting) myotubes (Fig 2B). A schematic of the experimental design is shown in Fig 2C. The timing for the application of PBM was 24hrs and immediately prior to EPS (2 doses of PBM in total). Non-contracting myotubes were also placed in the incubator for 3hrs as resting controls. Immediately after the 3hr EPS, intracellular levels of ATP and lactate secretion into the supernatant was determined for resting (controls), contracting EPS and contracting PBM myotubes where the treated group was denoted as 810E. EPS resulted in a significant decline in intracellular ATP levels of 21% ± 28 and an increase in lactate secretion, 35% ± 10 when compared to resting controls (Fig 2D–E). 810nm pretreatment resulted in a 32% ± 27 increase in ATP levels, and a 20% ± 12 decline in lactate release, when compared to EPS group (Fig 2D–E). To confirm the decline in lactate secretion was not due to a reduction in metabolic state, we measured glucose uptake immediately after EPS and saw no differences between groups (Fig 2F). To determine if there were any differences in protein homeostasis or cytotoxicity between all groups we measured intracellular protein content and LDH leak into the cell culture supernatant indicative of cell damage. We observed no changes between all groups (Fig 2G–H).

### Myotubes treated with 810nm have reduced contractile motion and calcium release during contractions

Muscular fatigue occurs when muscle performance, i.e., fiber contractions are at maximal strength and lactate acid build ups in the muscle. As lactate secretion was attenuated in 810 pretreatments, we wanted to confirm changes in contractions of individual myotubes. Therefore, live imaging (brightfield and fluorescence) was performed to observe changes in contractile motion and calcium signaling which is an integral part in myofiber contraction-relaxation. In brightfield imaging, EPS myotubes had a greater motion upon contraction with a 9% ± 5 change in myotube width compared to when the myotube is relaxed. 810 nm treated myotubes had a smaller change in myotube width during contractions, 5% ± 3. This result suggests that myotubes pretreated with 810nm were contracting with less force (Fig 3A). To confirm the changes in contractile motion, we also determined cytosolic levels of calcium in myotubes when they were in a relaxed state versus in contracted state as calcium plays an important role in sarcomere contraction in myotubes. We detected reduced cytosolic calcium upon contraction in 810nm pretreated myotubes compared to EPS myotubes (Fig 3B). These findings suggest that the decline in lactate secretion in 810 nm pretreatment contracting myotubes is due to a decline in contractile properties of the myotubes.

### 810nm prolongs spontaneous contractions of myotubes and an increase in mitochondrial proteins after EPS treatment

Although the EPS stimulated contractile motion was reduced for 810nm pretreated myotubes, the intracellular levels of ATP were increased compared to EPS alone (Fig 2D). ATP levels are highly conserved in cells and to maintain a basal state certain cellular pathways become activated. In muscle cells, contraction is an ATP dependent process, therefore, we wanted to determine spontaneous myotubes contractions and expression of mitochondrial proteins in the 810nm pretreated myotubes. After 3hrs of electric stimulation, we removed the carbon electrodes from the cell culture plate and allowed the myotubes to recover for 30minutes. For 810nm we saw an increased frequency (42% ± 21) of spontaneous post-EPS contraction (Fig 3C). To assess whether those physical changes are also detectable on the molecular level we isolated protein immediately after EPS stimulation. We determined changes in mitochondrial import machinery with translocase outer membrane subunit 70 (TOM70) and mitochondrial electron transport chain with succinate dehydrogenase subunit A (SDHA). TOM70 was increased 1.4 ± 0.3 fold in 810nm pretreated contracting myotubes compared to control contracting myotubes. SDHA, a subunit for complex 2 in the electron transport chain in the inner membrane of the mitochondria, was increased with 810nm pretreatment by 1.7 ± 0.3 fold (Fig 4A). To visualize mitochondrial matrix in myotubes, we did immunocytochemistry for citrate synthase (CS), an enzyme in the tricarboxylic acid (TCA) cycle present in the mitochondria and SDHA. Mitochondria are present along the length of myotubes; however, they are more dominant near the periphery of the nucleus. We observe a strong signal for both CS and SDH for 810nm treated myotubes when compared to EPS myotubes (Fig 4B). We counted the number of fibers positive for CS and SDHA and normalized to total number of myotubes present in the field. CS positive myotubes were increased 1.3 ± 0.2 and SDHA 1.4 ± 0.2 fold in 810nm pretreated contracting myotubes when compared to contracting myotubes (Fig 4C).

## Discussion

Our study suggests that there are beneficial effects seen in exercising muscle with 810 nm. To our knowledge, this is the only detailed study on 810 nm in contracting active myotubes. Clinical PBM treatment for muscular function often utilizes several wavelengths in combination [3] and future experiments need to be performed to fully understand the effect specific wavelengths (individually or in combination) may have on the tissue.

In resting myotubes that were not contracting, 810 nm increased ATP production 24 hours post treatment, and a similar increase was observed when myotubes were stimulated to contract. Along with higher ATP in actively contracting myotubes, 810 nm attenuated lactate secretion, altered contractile motion of individual myofibers and increased all mitochondrial proteins tested. There have been several studies in C2C12 myotubes [8,9] however studies on the effect of NIR treatment in contracting myotubes are limited with only one study in electrically stimulated myotubes. The study focused on induction of tetanic contractions resulting in mitochondrial dysfunction usually occurring in high intensity exercise [19]. In our study, the protocol for electrical stimulation was a frequency of 2 Hz with a 5msec pulse duration that lasted for 3 hrs and myotubes visually underwent twitch contractions. We were able to induce myotube anaerobic respiration by assessing biochemical markers such as ATP and lactate. We saw a decline in ATP and increase in lactate secretion in the contracting myotubes. 810 nm treatment attenuated the changes in both ATP and lactate secretion. The increase in ATP with 810 nm treatment might be attributed to mitochondrial activation. We observed increase in proteins in the mitochondrial outer membrane import machinery (Tom 70) and metabolic markers found in the mitochondria that are key players for the citric acid cycle (CS) and electron transport chain (SDH A) involved in oxidative phosphorylation for ATP production. The effect of 810 nm on mitochondrial signaling pathway [8] and ATP production [9] has been previously reported. The decline in lactate secretion with 810 nm could be partially attributed to a shift towards oxidative metabolism via mitochondrial activation as well as specific changes in contractile motion of individual myotubes. We observed that treated myotubes had reduced motion and intracellular calcium release upon contraction which is an indirect measure of ATP turnover and force production. The combinative effect of a lower ATP turnover and mitochondrial activation could have resulted in increased spontaneous contractions.

The favored wavelengths used by researchers and health professionals in PBM for muscular fatigue are in the red (630–660 nm) and near-infra red (808–980 nm) spectra. Light in this range can penetrate deeply into the tissue with a higher penetration depth for NIR wavelengths [3]. A study observed a similar effect of two different wavelengths, 660 or 830nm, in prevention of fatigue of healthy exercising individuals [20]. Possibly, due to the beneficial effect seen in the skeletal muscle system across all wavelengths in the range of 630–980 nm, there is an increase in availability of mixed red and NIR LED clusters and arrays that are used for PBM [3]. The combination of multiple wavelengths makes it difficult to optimize the energy delivered per wavelength. A previous study saw a difference in total power delivered for 780 nm and 850 nm in muscle cells where 850 nm required higher power [21]. Similarly in another study in skin, of the two wavelengths studied 630 and 850 nm, 850 nm required a higher energy [22]. Currently there is no consensus for PBM parameters as there are a large variety of commercially available devices. There is a range of total power used, time of PBM treatment, total energy delivered to the tissue, energy and power density. A review attempted to evaluate the PBM parameters used in vitro and in vivo research and determined that tissues with enriched mitochondria (i.e., muscle, brain, heart) require less energy than tissues with fewer mitochondria (skin, tendon, cartilage) [23]. A small number of studies were included in the review and future research is needed to confirm whether total energy delivered is dependent on tissue types as well as characterization of other PBM parameters.

The most common cell line for *in vitro* muscle research is C2C12 myoblasts that are differentiated to form myotubes. However, compared to adult myofibers in *in vivo* muscle tissue, C2C12 myotubes are immature. C2C12 myotubes have misaligned sarcomeres, reduced myofilament protein content and decline in transcripts involved in muscle maturation [24]. After myoblasts reach confluency, media is switched to low serum to induce differentiation. Myotubes can only remain attached to the surface of the cell culture dish for 7–10 days, after which they detach from the bottom. Due to this limitation, *in vitro* myotubes cannot differentiate for longer periods [25]. Along with the above-mentioned limitation of culturing adult myotubes, C2C12 myotubes also have myoblasts present that remain undifferentiated. This heterogenous population of cells makes it difficult to determine the effect of treatment on a specific cell type. There are also major differences across *in vitro* muscle cell lines from mouse, rat and human [26]. Additionally, cells in dish lack the motor nerve, blood supply, circulating hormones and external stimulus such as stretch/force of the muscle. These factors maintain skeletal muscle health and cannot be recapitulated in an *in vitro* model. Therefore, future work is necessary to fully understand the effect of photobiomodulation in mouse models of exercise.

In conclusion, 810 nm wavelength increased ATP in resting myotubes and altered the metabolic phenotype of actively contracting myotubes with an increase in ATP and a decline in secreted lactate. Additionally, treatment physically altered contractile motion and had a specific upregulation of mitochondrial proteins in myotubes. These finding suggest that 810 nm treatment altered metabolic and physical properties of contracting myotubes due to mitochondrial activation. Furthermore, future studies are required to fully understand the biological responses activated by various wavelengths and the effect on muscle performance.

## Supporting information

S1 Fig Representative raw data from capillary Western immune assay from ProteinSimple. The Y axis represents chemiluminescence signal and x axis represents molecular weight of protein. The 46–48 kDa potein in b-actin (A and B) and 60 kDa protein is Tom70 (A) and SDH A (B).
(EPS)

S1 File Raw data for the manuscript. (XLSX)

## References

[1] Hamblin MR. Mechanisms and mitochondrial redox signaling in photobiomodulation. Photochem Photobiol. 2018;94(2):199–212. doi: 10.1111/php.12864 29164625 PMC5844808

[2] Rossato M, Dellagrana RA, Sakugawa RL, Baroni BM, Diefenthaeler F. Dose-response effect of photobiomodulation therapy on muscle performance and fatigue during a multiple-set knee extension exercise: a randomized, crossover, double-blind placebo-controlled trial. Photobiomodul Photomed Laser Surg. 2020;38(12):758–65. doi: 10.1089/photob.2020.4820 33232629

[3] Ferraresi C, Huang Y-Y, Hamblin MR. Photobiomodulation in human muscle tissue: an advantage in sports performance?. J Biophotonics. 2016;9(11–12):1273–99. doi: 10.1002/jbio.201600176 27874264 PMC5167494

[4] Macedo AB, Moraes LHR, Mizobuti DS, Fogaça AR, Moraes FDSR, Hermes T de A, et al. Low-level laser therapy (LLLT) in dystrophin-deficient muscle cells: effects on regeneration capacity, inflammation response and oxidative stress. PLoS One. 2015;10(6):e0128567. doi: 10.1371/journal.pone.0128567 26083527 PMC4470633

[5] Nakano J, Kataoka H, Sakamoto J, Origuchi T, Okita M, Yoshimura T. Low-level laser irradiation promotes the recovery of atrophied gastrocnemius skeletal muscle in rats. Exp Physiol. 2009;94(9):1005–15. doi: 10.1113/expphysiol.2009.047738 19525315

[6] de Oliveira AR, da Silva FS, Bortolin RH, Marques DEDS, Ramos GV, Marqueti RC, et al. Effect of photobiomodulation and exercise on early remodeling of the Achilles tendon in streptozotocin-induced diabetic rats. PLoS One. 2019;14(2):e0211643. doi: 10.1371/journal.pone.0211643 30716140 PMC6361457

[7] Bertin JSF, Marques MJ, Macedo AB, de Carvalho SC, Neto HS. Effect of Photobiomodulation on denervation-induced skeletal muscle atrophy and autophagy: a study in mice. J Manipulative Physiol Ther. 2022;45(2):97–103. doi: 10.1016/j.jmpt.2022.03.011 35753870

[8] Nguyen LM-D, Malamo AG, Larkin-Kaiser KA, Borsa PA, Adhihetty PJ. Effect of near-infrared light exposure on mitochondrial signaling in C2C12 muscle cells. Mitochondrion. 2014;14(1):42–8. doi: 10.1016/j.mito.2013.11.001 24246911

[9] Ferraresi C, Kaippert B, Avci P, Huang Y-Y, de Sousa MVP, Bagnato VS, et al. Low-level laser (light) therapy increases mitochondrial membrane potential and ATP synthesis in C2C12 myotubes with a peak response at 3-6 h. Photochem Photobiol. 2015;91(2):411–6. doi: 10.1111/php.12397 25443662 PMC4355185

[10] Carter S, Solomon TPJ. In vitro experimental models for examining the skeletal muscle cell biology of exercise: the possibilities, challenges and future developments. Pflugers Arch. 2019;471(3):413–29. doi: 10.1007/s00424-018-2210-4 30291430

[11] Nedachi T, Fujita H, Kanzaki M. Contractile C2C12 myotube model for studying exercise-inducible responses in skeletal muscle. Am J Physiol Endocrinol Metab. 2008;295(5):E1191–204. doi: 10.1152/ajpendo.90280.2008 18780777

[12] Manabe Y, Miyatake S, Takagi M, Nakamura M, Okeda A, Nakano T, et al. Characterization of an acute muscle contraction model using cultured C2C12 myotubes. PLoS One. 2012;7(12):e52592. doi: 10.1371/journal.pone.0052592 23300713 PMC3534077

[13] Nikolic N, Gorgens SW, Thoresen GH, Aas V, Eckel J, Eckardt K. Electrical pulse stimulation of cultured skeletal muscle cells as a model for in vitro exercise - possibilities and limitations. Acta Physiol (Oxf). 2017;220(3):310–31. doi: 10.1111/apha.12830 27863008

[14] Burch N, Arnold A-S, Item F, Summermatter S, Brochmann Santana Santos G, Christe M, et al. Electric pulse stimulation of cultured murine muscle cells reproduces gene expression changes of trained mouse muscle. PLoS One. 2010;5(6):e10970. doi: 10.1371/journal.pone.0010970 20532042 PMC2881042

[15] Cuenda A, Cohen P. Stress-activated protein kinase-2/p38 and a rapamycin-sensitive pathway are required for C2C12 myogenesis. J Biol Chem. 1999;274(7):4341–6. doi: 10.1074/jbc.274.7.4341 9933636

[16] Gerhardt KP, Olson EJ, Castillo-Hair SM, Hartsough LA, Landry BP, Ekness F, et al. An open-hardware platform for optogenetics and photobiology. Sci Rep. 2016;6:35363. doi: 10.1038/srep35363 27805047 PMC5096413

[17] Schneider CA, Rasband WS, Eliceiri KW. NIH image to ImageJ: 25 years of image analysis. Nat Methods. 2012;9(7):671–5. doi: 10.1038/nmeth.2089 22930834 PMC5554542

[18] Dompe C, Moncrieff L, Matys J, Grzech-Leśniak K, Kocherova I, Bryja A, et al. Photobiomodulation-underlying mechanism and clinical applications. J Clin Med. 2020;9(6):1724. doi: 10.3390/jcm9061724 32503238 PMC7356229

[19] Xu X, Zhao X, Liu TC-Y, Pan H. Low-intensity laser irradiation improves the mitochondrial dysfunction of C2C12 induced by electrical stimulation. Photomed Laser Surg. 2008;26(3):197–202. doi: 10.1089/pho.2007.2125 18484910

[20] de Almeida P, Lopes-Martins RAB, De Marchi T, Tomazoni SS, Albertini R, Corrêa JCF, et al. Red (660 nm) and infrared (830 nm) low-level laser therapy in skeletal muscle fatigue in humans: what is better? Lasers Med Sci. 2012;27(2):453–8. doi: 10.1007/s10103-011-0957-3 21814736 PMC3282894

[21] Lovisetto R, Malavazzi TCDS, Andreo L, Rodrigues MFSD, Bussadori SK, Fernandes KPS, et al. Photobiomodulation using different infrared light sources promotes muscle precursor cells migration and proliferation. Photonics. 2022;9(7):469. doi: 10.3390/photonics9070469

[22] Camargo CP, Forner-Cordero A, Silva BM, de Souza VM, Cunha HS, de Oliveira Feitosa Y, et al. Effect of photobiomodulation with different wavelengths on radiodermatitis treatment. Plast Reconstr Surg Glob Open. 2023;11(2):e4809. doi: 10.1097/GOX.0000000000004809 36751505 PMC9894341

[23] Zein R, Selting W, Hamblin MR. Review of light parameters and photobiomodulation efficacy: dive into complexity. J Biomed Opt. 2018;23(12):1–17. doi: 10.1117/1.JBO.23.12.120901 30550048 PMC8355782

[24] Denes LT, Riley LA, Mijares JR, Arboleda JD, McKee K, Esser KA, et al. Culturing C2C12 myotubes on micromolded gelatin hydrogels accelerates myotube maturation. Skelet Muscle. 2019;9(1):17. doi: 10.1186/s13395-019-0203-4 31174599 PMC6555731

[25] Schiaffino S, Rossi AC, Smerdu V, Leinwand LA, Reggiani C. Developmental myosins: expression patterns and functional significance. Skelet Muscle. 2015;5:22. doi: 10.1186/s13395-015-0046-6 26180627 PMC4502549

[26] Abdelmoez AM, Sardón Puig L, Smith JAB, Gabriel BM, Savikj M, Dollet L, et al. Comparative profiling of skeletal muscle models reveals heterogeneity of transcriptome and metabolism. Am J Physiol Cell Physiol. 2020;318(3):C615–26. doi: 10.1152/ajpcell.00540.2019 31825657 PMC7099524

